# Supplementary figures and images for: Staphylococcus aureus viewed from the perspective of 40,000+ genomes
Source: PeerJ. 2018 Jul 12;6:e5261. doi: 10.7717/peerj.5261 (PMC6046195; doi:10.7717/peerj.5261)

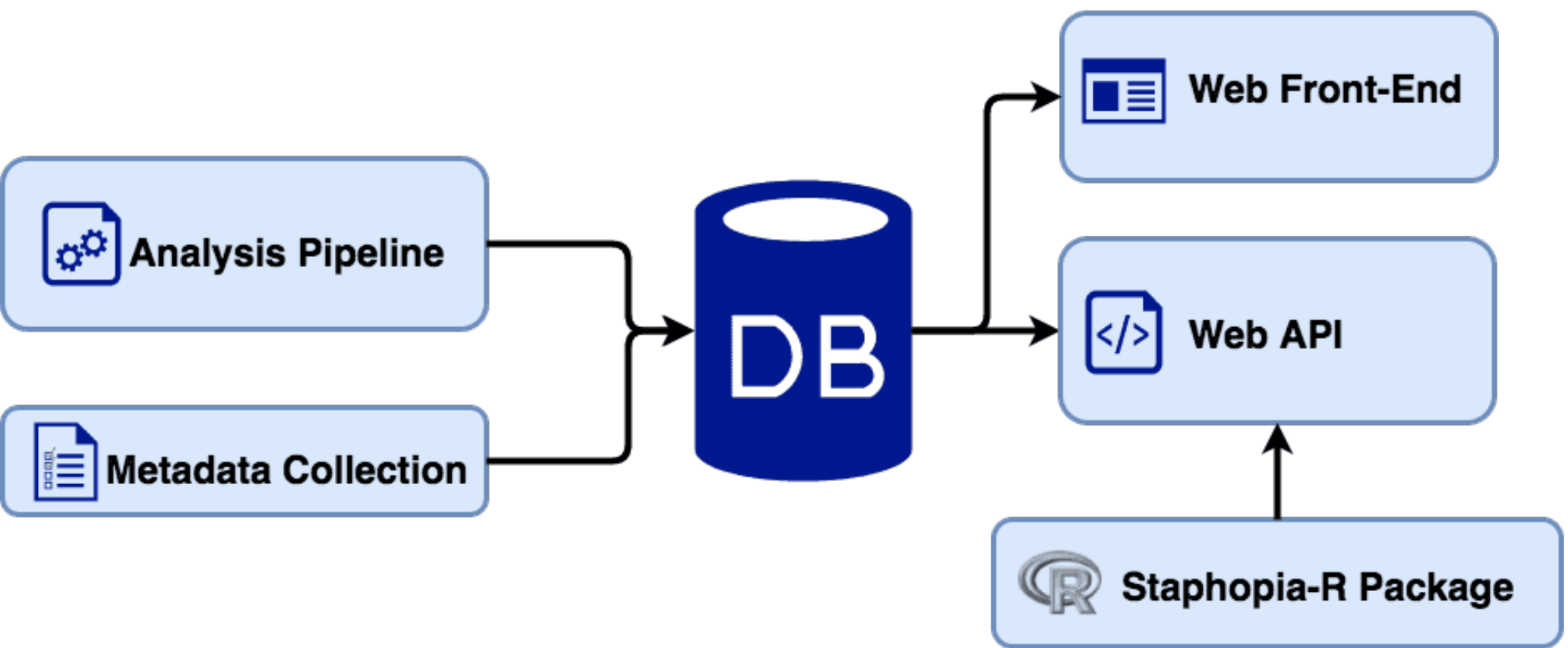

Supplement: Supplemental Information 1 — This figure provides an overview of Staphopia. Samples are processed by the analysis pipeline and stored in the database. Metadata collected from linked publications are also stored in the database. This information is then made available through a web front-end or a web application programming interface (API). An R package has also been developed to programmatically access the web API. [file peerj-06-5261-s001.pdf]

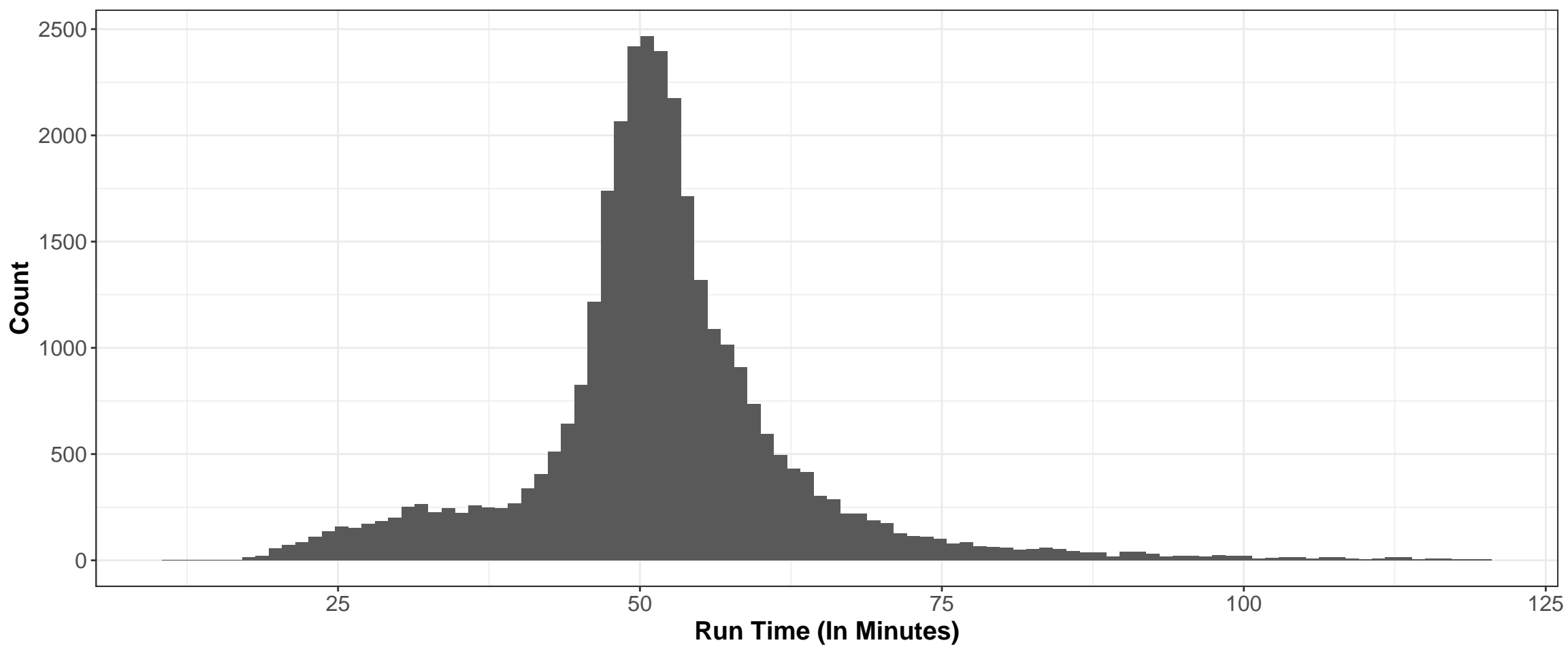

Supplement: Supplemental Information 2 — Overall run time statistics were available for 31,587 of the completed CGC jobs. Mean run time was 51 minutes (median 52 minutes). There were 983 jobs that took more than 80 minutes to complete. [file peerj-06-5261-s002.pdf]

Tree scale: 0.001

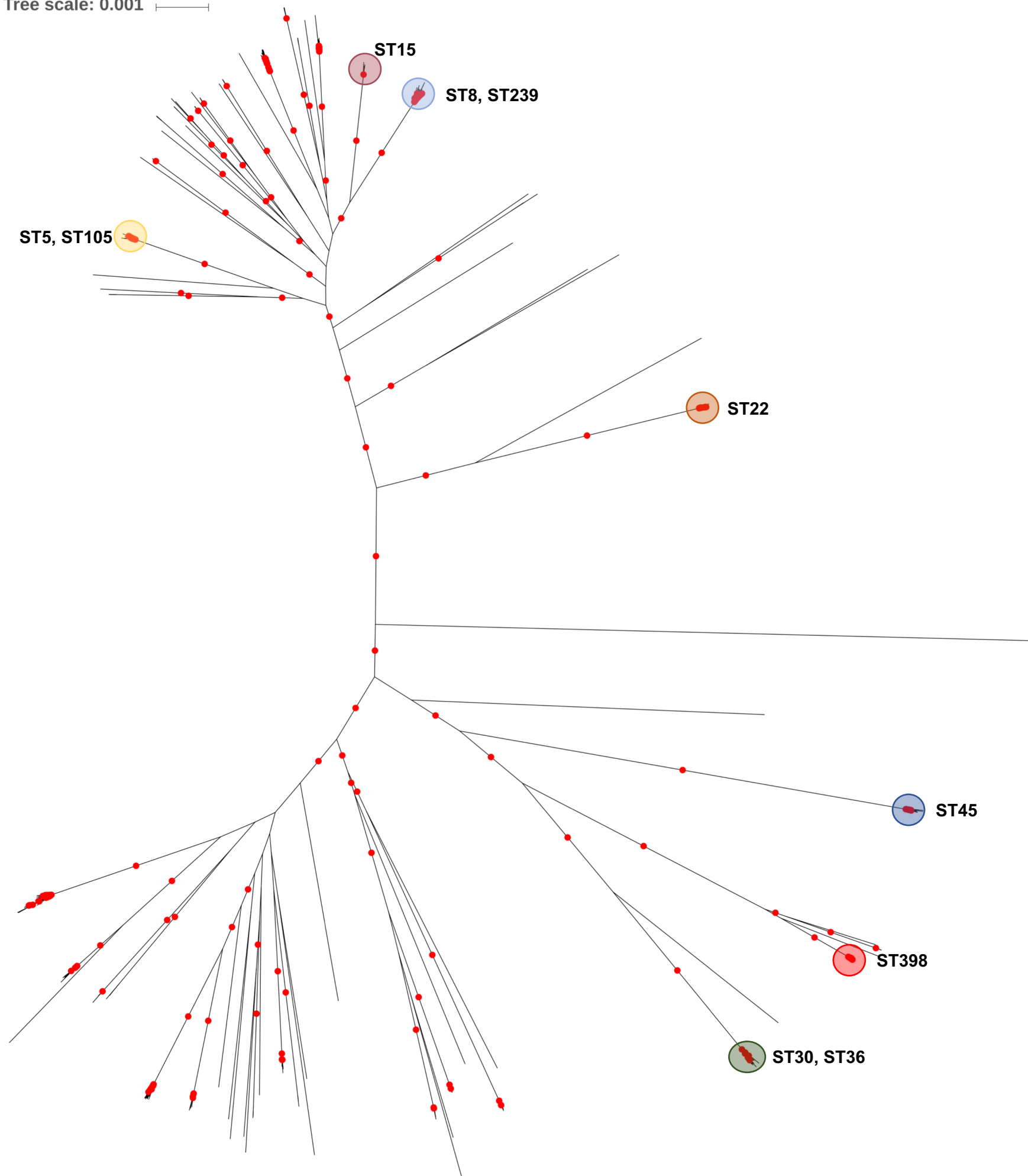

Supplement: Supplemental Information 3 — An unrooted phylogenetic representation of the 380 genome non-redundant set (one representative per ST, all published and gold rank) using IQ-Tree (Nguyen et al., 2015). The putatively recombinant positions predicted using ClonalFrameML (Didelot & Wilson, 2015) were removed from the alignment. Clonal complexes containing the top ten most common STs are indicated with colored circles. The tree was built from 878 reconstructed core genes (please see Methods section) with 44,377 sites. Branches supported with probability > 0.9 are marked by red dots. The likelihood score for the tree was −1,890,510. [file peerj-06-5261-s003.pdf]
